# Patient-reported gastrointestinal symptoms in patients with peritoneal dialysis: the prevalence, influence factors and association with quality of life

**DOI:** 10.1186/s12882-022-02723-9

**Published:** 2022-03-09

**Authors:** Chunyan Yi, Xin Wang, Hongjian Ye, Jianxiong Lin, Xiao Yang

**Affiliations:** Department of Nephrology, The First Affiliated Hospital, Sun Yat-sen University and Key Laboratory of Nephrology, Ministry of Health, 58th, Zhongshan Road II, Guangzhou, 510080 Guangdong Province China

**Keywords:** Peritoneal dialysis, Gastrointestinal symptoms, Quality of life

## Abstract

**Background:**

The aims of this study were to investigate the prevalence and the influence factors of gastrointestinal symptoms, and its association with the quality of life (QOL) in peritoneal dialysis (PD) patients.

**Methods:**

Continuous ambulatory PD patients (CAPD) who followed up in our PD center between March 2016 and December 2017 were enrolled in this cross-sectional study. Gastrointestinal symptom rating scale (GSRS) was used to evaluate gastrointestinal symptoms. The related clinical data were also collected. Multiple linear regression analysis was test for the influence factors associated with score of GSRS and QOL.

**Results:**

This study included 471 CAPD patients. The mean age was 48.5±13.9 years, 53.9% were male and 15.1% with diabetic nephropathy. The median duration of PD was 37.3 (17.5~66.5) months. The median score of GSRS was 1.2(1.1~1.3) scores. Totally 82.2% (*n*=387) CAPD patients had at least one gastrointestinal symptom. Higher glycosylated hemoglobin, higher score of depression, lower diastolic blood pressure, urine output, score of instrumental activities of daily living scale and more amount of pills per day were independently associated with higher score of GSRS (all *P*<0.05). Score of dyspepsia and eating dysfunction were independently associated with worse score of QOL and physical health (all *P*<0.05).

**Conclusions:**

Gastrointestinal symptoms were common but not serious in CAPD patients. Glycemic control, depression, blood pressure, urine output, activity of daily life and amount of pills were all associated with gastrointestinal symptoms. Moreover, gastrointestinal symptoms were correlated with QOL of PD patients.

End stage renal disease (ESRD) is a complex disease. Gastrointestinal symptoms related to uraemia, medications and other comorbidities are common in patients with ESRD. Endoscopic evaluation and mucosal biopsies are ideal ways to diagnose gastrointestinal diseases, but they are difficult to be widely used in peritoneal dialysis (PD) patients because of their invasiveness. There are several scales used to evaluate functional gastrointestinal symptoms in dialysis patients, such as Rome I, Rome II, Rome III and gastrointestinal symptom rating scale (GSRS). The prevalence of gastrointestinal symptoms in patients with PD evaluated by different assessment scales varied greatly, which ranged from 14.2% to 90.3% [1–5].

The underlying pathogenesis of gastrointestinal symptoms in PD patients has not been clearly elucidated. The evidence base of gastrointestinal symptoms in dialysis patients was limited, and further investigation of preventable causes and potential interventions were required in future research [6]. Since dextrose dialysate was indwelled into the peritoneal cavity of PD patients, the particularity of PD treatment might affect gastrointestinal function through delayed gastric emptying [7–10], increased intraperitoneal pressure and decreased lower esophageal sphincter pressure [11, 12]. It is necessary to further study the influence factors of gastrointestinal symptoms in PD patients. Moreover, gastrointestinal symptoms may play an important role on quality of life (QOL) of PD patients. Although previous studies have explored the relationship between gastrointestinal symptoms and QOL in dialysis patients, these studies only used univariate analysis that did not adjust for other confounding factors [4, 13, 14]. Therefore, the aims of this study were to investigate the prevalence and the influence factors of gastrointestinal symptoms in PD patients, and to determine the association of gastrointestinal symptoms with QOL.

## Materials and methods

### Participants

This cross-sectional study enrolled ESRD patients who received PD treatment and followed up in a single PD center in Southern China from March 2016 to December 2017. Inclusion criteria were as following: at least 18 years old, receiving continuous ambulatory PD (CAPD) treatment more than 3 months. Patients with severe gastrointestinal diseases (digestive tract hemorrhage, recurrent peptic ulcers, gastrointestinal perforation, intestinal obstruction, gastrointestinal malignant tumors and gastrointestinal surgery, etc.), or severe infection (refractory peritonitis, septic shock, etc.) during the past 3 months, or other complications (malignant tumor, cardiovascular disease requiring hospitalization, etc.), or inability to complete the investigation, or unwillingness to participate this study were excluded. All participants used the conventional PD solutions (Dianeal 1.5%, 2.5%, or 4.25% dextrose; Baxter Healthcare, Guangzhou, China). Y sets and twin bag systems were used in all participants. This study was approved by the Human Ethics Committee of Sun Yat-sen University [Ethics Review (2016) NO.215] and the written informed consent of patients was obtained.

### *Measurement of* gastrointestinal *symptoms*

Gastrointestinal symptoms of participants were assessed by GSRS. The original GSRS included 15 items, which could be grouped into five dimensions: abdominal pain (three items), reflux (two items), dyspepsia (four items), diarrhea (three items) and constipation syndrome (three items) [15]. Eating dysfunction syndrome, which was developed by Svedlund [16], was added into the original GSRS. It included three items: early satiety, difficulties in eating normal portions, and postprandial pain. All items used a 7-grade Likert scale defined by descriptive anchors (1=none, 2=minor, 3=mild, 4=moderate, 5=moderately severe, 6=severe, 7=very severe discomfort). The questions concerned symptom severity during the previous two weeks. Each dimension score was calculated as the mean value of the items belonging to the specific syndrome with a value from 1 to 7.

### Measurements of activity of daily living, depression and quality of life

Activity of daily living was assessed by Barthel index [17] and instrumental activities of daily living scale (IADLs) [18]. The Chinese version of Beck Depression Inventory-II was used to evaluate the severity of depressive symptoms [19]. QOL was evaluated by the Short Form of Medical Outcomes Study (SF-36) [20]. It was a self-administered 36-item questionnaire, which was divided into eight dimensions including physical functioning, role-physical, bodily pain, general health status, vitality, social functioning, role-emotional, and mental health. The eight dimensions could be divided into two components, which were the physical component scale including the first four dimensions, and the mental component scale including the remaining four dimensions. Each dimension was scored from 0 to 100. Higher scores of the scale indicated the better QOL. Based on the reference, total score of QOL was arithmetic averaging of the eight SF-36 domains scores [21].

### Data collection

Demographic, clinical and laboratory data were obtained when participants were enrolled in the study. Demographic data included age, gender and primary renal disease. Clinical data included duration of PD, PD dose, dwell volume of dialysate, urine output, blood pressure, body mass index and total amount of pills per day. Laboratory data included hemoglobin, high-sensitivity C-reactive protein, serum albumin, serum calcium, serum phosphorus, intact parathyroid hormone, total cholesterol, triglycerides, serum sodium, serum potassium, glycosylated hemoglobin, blood urea nitrogen and serum creatinine and clearance index of urea (Kt/V). Charlson comorbidity index [22] was used to assess comorbidities of participants.

### Statistical analysis

All statistical analyses were performed by SPSS Statistics 19.0. Continuous variables of approximately normally distributed were expressed as mean (standard deviation) and compared with independent *t* test. Skewed continuous variables were expressed as median and interquartile range and compared with Mann-Whitney test. Categorical variables were expressed as number and percentage and compared with chi-square test. Multiple linear regression analysis was test for the influence factors that associated with score of GSRS and QOL with significance level of selection entry at 0.05. Results were considered significant when double side *P*<0.05.

## Results

### Overall patient situation

**Fig. 1:**
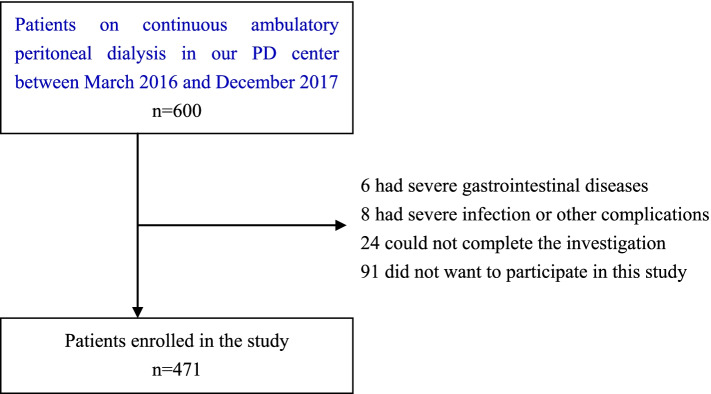
Flow chart

**Fig. 2:**
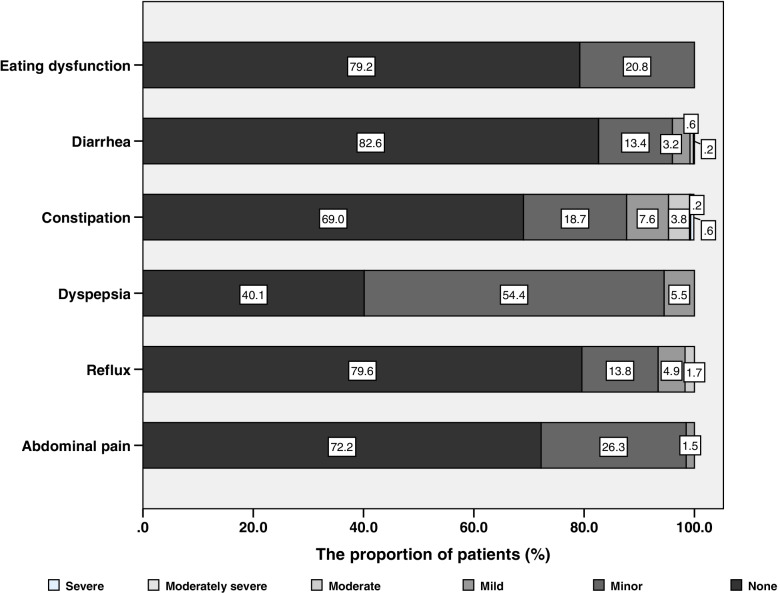
Prevalence and severity of gastrointestinal symptoms reported by peritoneal dialysis patients

A total of 600 CAPD patients were recruited during the study period. Among them, six patients had severe gastrointestinal diseases, eight patients had severe infection or other complications, 24 patients could not complete the investigation and 91 patients did not want to participate in this study (Fig. 1). Finally, 471 CAPD patients were included in this study. The mean age was 48.5±13.9 years, and the median duration of PD was 37.3 (17.5~66.5) months. Totally 53.9% (*n*=254) patients were male, and 15.1% (*n*=71) patients were diabetic nephropathy. The median Charlson comorbidity index was 3.0 (2.0~4.0) scores. A total of 387(82.2%) CAPD patients developed at least one gastrointestinal symptom in this study. Among them, 59.9% (*n*=282) patients had dyspepsia, 31.0% (*n*=146) patients had constipation, 27.8% (*n*=131) patients had abdominal pain, 20.8% (*n*=98) patients had eating dysfunction, 20.4% (*n*=96) patients had reflux and 17.4% (*n*=82) patients had diarrhea. The severity of each dimension was showed in Fig. 2. Compared with patients without gastrointestinal symptom, patients with gastrointestinal symptoms had lower urine output, higher depression score and higher proportion of use of proton pump inhibitor drugs. (all *P*<0.05) (Table 1).

**Table 1:** The demographic and clinical data of all peritoneal dialysis patients

Variables	Total (*n*=471)	Non-gastrointestinal symptom group (*n*=84)	Gastrointestinal symptoms group (*n*=387)	*P* values
Age (years)	48.5±13.9	47.5±13.6	48.7±13.9	0.481
Male (n, %)	254(53.9%)	51(60.7%)	203(52.5%)	0.169
Primary renal disease (n, %)				0.173
Glomerulonephritis	307(65.2%)	59(70.3%)	248(64.1%)	
Diabetic nephropathy	71(15.1%)	8(9.5%)	63(16.3%)	
Renal vascular diseases	39(8.3%)	10(11.9%)	29(7.5%)	
Other	54(11.5%)	7(8.3%)	47(12.1%)	
Charlson comorbidity index (score)	3.0(2.0~4.0)	3.0(2.0~4.0)	3.0(2.0~5.0)	0.096
Duration of peritoneal dialysis (months)	37.3(17.5~66.5)	36.4(19.1~60.7)	38.0(16.7~67.5)	0.697
Total amount of pills per day (number)	14.0(9.0~21.0)	14.3(8.5~22.9)	14.0(9.0~21.0)	0.730
Use of proton pump inhibitor drugs (yes)	36(7.6%)	1(1.2%)	35(9.0%)	0.026
Use of gastrointestinal motility drugs (yes)	18(3.8%)	1(1.2%)	17(4.4%)	0.283
Use of phosphorus binders (yes)	195(41.4%)	30(35.7%)	165(42.6%)	0.243
Urine output (ml/d)	300.0(0.0~800.0)	540.0(15.0~1090.0)	250.0(0.0~700.0)	0.025
Peritoneal dialysis dose (ml/d)	8000.0(8000.0~10000.0)	8000.0(8000.0~10000.0)	8000.0(8000.0~10000.0)	0.776
Dwell volume of dialysate (ml)	2000.0(2000.0~2000.0)	2000.0(2000.0~2000.0)	2000.0(2000.0~2000.0)	0.114
Systolic blood pressure (mmHg)	135.8±22.1	137.7±19.5	135.4±22.6	0.383
Diastolic blood pressure (mmHg)	83.2±13.5	85.6±12.6	82.7±13.6	0.073
Body mass index (kg/m^2^)	22.2±3.2	22.5±3.3	22.1±3.2	0.395
Hemoglobin (g/L)	113.2±19.7	111.5±19.9	113.6±19.6	0.376
High-sensitivity C-reactive protein (mg/L)	1.6(0.6~5.0)	1.5(0.6~3.7)	1.7(0.6~5.3)	0.884
Serum albumin (g/L)	37.0±4.1	37.6±3.6	36.9±4.2	0.126
Serum calcium (mmol/L)	2.3±0.2	2.3±0.2	2.3±0.2	0.667
Serum phosphorus (mmol/L)	1.6±0.5	1.6±0.5	1.6±0.5	0.842
Intact parathyroid hormone (pg/ml)	330.7(157.6~651.7)	371.4(137.7~654.0)	328.9(160.4~651.7)	0.760
Total cholesterol (mmol/L)	4.9(4.2~5.7)	4.9(4.1~5.4)	4.9(4.2~5.7)	0.437
Triglycerides (mmol/L)	1.5(1.1~2.2)	1.5(1.0~2.4)	1.5(1.1~2.2)	0.878
Serum sodium (mmol/L)	138.3±3.9	138.3±3.0	138.3±4.1	0.907
Serum potassium (mmol/L)	4.1±0.7	4.2±0.6	4.1±0.7	0.350
Glycosylated hemoglobin (mmol/mol)	37.5±11.5	36.9±10.7	37.7±11.7	0.603
Blood urea nitrogen (mmol/L)	16.8(14.4~20.4)	17.1(14.3~21.1)	16.8(14.4~20.3)	0.550
Serum creatinine (umol/L)	1004(798~1223)	975.0(804.8~1339.0)	1006.0(795.0~1207.0)	0.560
Clearance index of urea	2.1(1.8~2.4)	2.2(1.7~2.5)	2.1(1.8~2.4)	0.356
Barthel index (score)	99.1±4.6	98.5±7.9	99.3±3.5	0.387
Instrumental activities of daily living scale (score)	22.7±3.8	22.8±4.2	22.6±3.7	0.772
Depression (score)	10.0(5.0~16.0)	8.0(4.0~14.8)	10.0(5.0~17.0)	0.033
Gastrointestinal symptom rating scale (score)	1.2(1.1~1.3)	-	1.2(1.1~1.4)	-
Abdominal pain (score)	1.0(1.0~1.3)	-	1.0(1.0~1.3)	-
Reflux (score)	1.0(1.0~1.0)	-	1.0(1.0~1.0)	-
Dyspepsia (score)	1.3(1.0~1.5)	-	1.3(1.0~1.5)	-
Constipation (score)	1.0(1.0~1.3)	-	1.0(1.0~1.7)	-
Diarrhea (score)	1.0(1.0~1.0)	-	1.0(1.0~1.0)	-
Eating disfunction (score)	1.0(1.0~1.0)	-	1.0(1.0~1.3)	-
Total score of quality of life (score)	60.0±17.8	60.6±19.2	59.9±17.5	0.724
Physical Component Scale (score)	57.0±18.4	58.0±19.8	56.8±18.0	0.585
Mental Component Scale (score)	63.0±19.7	63.3±20.6	63.0±19.5	0.897

### Influence factors of gastrointestinal symptoms

Since GSRS was a continuous variable of skewness distribution, logarithmic transformation was performed for it. The unary linear regression analysis showed that age, Charlson comorbidity index, duration of PD, total amount of pills per day, PD dose, urine output, diastolic blood pressure, glycosylated hemoglobin, score of IADLs and depression were associated with the score of GSRS in PD patients (all *P*<0.05). These factors, gender and serum creatinine were include into the multiple linear regression analysis. The result showed that higher level of glycosylated hemoglobin, higher score of depression, lower urine output, lower diastolic blood pressure, lower score of IADLs and more amount of pills per day were independent influence factors associated with the higher score of GSRS after adjustment for confounders (all *P*<0.05) (Table 2).

**Table 2:** The influence factors of score of gastrointestinal symptom rating scale

Variables	Unary linear regression analysis		Multiple linear regression analysis	
	B (95% CI)	*P* values	B (95% CI)	*P* values
Age (years)	0.012(0.005~0.020)	0.001	-0.007(-0.018~0.004)	0.193
Male (yes)	-0.006(-0.021~0.008)	0.401	-0.005(-0.020~0.011)	0.545
Charlson comorbidity index (score)	0.016(0.009~0.023)	<0.001	-0.003(-0.017~0.010)	0.628
Duration of peritoneal dialysis (months)	0.007(0.00006~0.014)	0.048	-0.003(-0.010~0.005)	0.502
Total amount of pills per day (number)	0.013(0.006~0.020)	<0.001	0.008(0.0003~0.015)	0.043
Peritoneal dialysis dose (ml/d)	0.008(0.001~0.015)	0.025	0.001(-0.008~0.011)	0.790
Urine output (ml/d)	-0.016(-0.023~-0.009)	<0.001	-0.016(-0.026~-0.006)	0.002
Diastolic blood pressure (mmHg)	-0.019(-0.026~-0.012)	<0.001	-0.014(-0.021~-0.006)	<0.001
Glycosylated hemoglobin (mmol/mol)	0.015(0.008~0.023)	<0.001	0.011(0.002~0.019)	0.012
Serum creatinine (umol/L)	-0.004(-0.011~0.004)	0.328	-0.010(0-0.019~0.000)	0.050
Instrumental activities of daily living scale (score)	-0.017(-0.024~-0.010)	<0.001	-0.010(-0.018~-0.001)	0.023
Depression (score)	0.014(0.007~0.021)	<0.001	0.009(0.002~0.017)	0.009

*B* nonnormalized coefficient; *95% CI* 95% confidence interval

### *Correlation between* gastrointestinal *symptoms and QOL*

Using unary linear regression analysis, it was found that higher score of GSRS, reflux, dyspepsia and eating dysfunction were associated with lower total score of QOL in PD patients (all *P*<0.05). Moreover, advancing age [B=-1.786, 95% confidence interval (CI): -3.392~-0.181; *P*=0.029], longer duration of PD (B=-1.737, 95%CI: -3.343~-0.130; *P*=0.034), higher Charlson comorbidity index (B=-2.497, 95%CI: -4.095~-0.900; *P*=0.002), lower urine output (B=2.765, 95%CI: 1.171~4.359; *P*=0.001), diastolic blood pressure (B=2.520, 95%CI: 0.922~4.118; *P*=0.002), serum albumin (B=1.889, 95%CI: 0.284~3.494; *P*=0.021) and triglyceride (B=1.952, 95%CI: 0.349~3.556; *P*=0.017) were all associated with lower total score of QOL. After adjustment for confounders, it was found that higher score of dyspepsia and eating dysfunction were independently associated with lower total score of QOL (all *P*<0.05) (Table 3).

**Table 3:** The association of score of gastrointestinal symptom rating scale with total score of quality of life

Varibles	Model 1 B (95%CI)	Model 2 B (95%CI)	Model 3 B (95%CI)
Gastrointestinal symptom rating scale (score)	-2.287(-3.887~-0.686)**	-1.210(-2.880~0.460)	-1.390(-3.069~0.289)
Reflux (score)	-1.686(-3.293~-0.079)*	-1.152(-2.757~0.454)	-1.131(-2.736~0.473)
Dyspepsia (score)	-2.319(-3.919~-0.718)**	-1.919(-3.522~-0.316)*	-1.900(-3.485~-0.315)*
Eating dysfunction (score)	-2.935(-4.527~-1.343)***	-2.222(-3.859~-0.584)**	-1.894(-3.542~-0.246)*

Model 1: Unadjusted. Model 2: Adjusted for age, gender, duration of peritoneal dialysis, Charlson commorbidity index, urine output and diastolic blood pressure. Model 3: Model 2 + adjusted for serum albumin and triglyceride

*B* nonnormalized coefficient; *95% CI* 95% confidence interval

*P<0.05 **P<0.01 ***P<0.001

The unary linear regression analysis showed that higher score of GSRS, reflux, dyspepsia and eating dysfunction were significantly associated with lower score of physical component scale of PD patients (all *P*<0.05). Moreover, advancing age (B=-1.953, 95%CI: -3.608~-0.297; *P*=0.021), longer duration of PD (B=-2.004, 95%CI: -3.659~-0.348; *P*=0.018), higher Charlson comorbidity index (B=-2.699, 95%CI: -4.346~-1.051; *P*=0.001), lower urine output (B=2.976, 95%CI: 1.332~4.619; *P<*0.001), diastolic blood pressure (B=2.501, 95%CI: 0.851~4.151; *P*=0.003), serum albumin (B=2.273, 95%CI: 0.621~3.926; *P*=0.007) and triglyceride (B=2.034, 95%CI: 0.376~3.691; *P*=0.016) were significantly associated with lower score of physical component scale. After adjustment for confounders, higher score of dyspepsia and eating dysfunction were independently associated with lower score of physical component scale (all *P*<0.05) (Table 4).

**Table 4:** The association of score of gastrointestinal symptom rating scale with physical health

Varibles	Model 1 B (95%CI)	Model 2 B (95%CI)	Model 3 B (95%CI)
Gastrointestinal symptom rating scale (score)	-2.604(-4.253~-0.956)**	-1.509(-3.228~0.209)	-1.652(-3.384~0.080)
Reflux (score)	-2.045(-3.700~-0.390)*	-1.505(-3.157~0.146)	-1.512(-3.166~0.142)
Dyspepsia (score)	-2.768(-4.415~-1.122)**	-2.398(-4.044~-0.751)**	-2.362(-3.995~-0.730)**
Eating dysfunction (score)	-3.263(-4.901~-1.624)***	-2.505(-4.190~-0.821)**	-2.195(-3.893~-0.496)*

Model 1: Unadjusted. Model 2: Adjusted for age, gender, duration of peritoneal dialysis, Charlson commorbidity index, urine output and diastolic blood pressure. Model 3: Model 2 + adjusted for serum albumin and triglyceride

*B* nonnormalized coefficient; *95% CI* 95% confidence interval

*P<0.05 **P<0.01 ***P<0.001

Higher score of GSRS, dyspepsia, eating dysfunction and Charlson comorbidity index (B=-2.296, 95%CI: -4.073~-0.520; *P*=0.011), lower urine output (B=2.555, 95%CI: 0.781~4.328; *P*=0.005), diastolic blood pressure (B=2.539, 95%CI: 0.766~4.313; *P*=0.005) and triglyceride (B=1.871, 95%CI:0.093~3.649; *P*=0.039) were associated with lower score of mental component scale of PD patients by using unary linear regression analysis. After adjustment for other confounders, the score of GSRS and each dimension were not associated with lower score of mental component scale (all *P*>0.05) (Table 5).

**Table 5:** The association of score of gastrointestinal symptom rating scale with mental health

Varibles	Model 1 B (95%CI)	Model 2 B (95%CI)	Model 3 B (95%CI)
Gastrointestinal symptom rating scale (score)	-1.969(-3.749~-0.189)*	-0.912(-2.776~0.952)	-1.087(-2.946~0.773)
Dyspepsia (score)	-1.869(-3.650~-0.088)*	-1.401(-3.190~0.387)	-1.390(-3.158~0.378)
Eating dysfunction (score)	-2.607(-4.380~-834)**	-1.954(-3.783~-0.126)*	-1.569(-3.395~0.257)

Model 1: Unadjusted. Model 2: Adjusted for age, gender, Charlson commorbidity index, urine output and diastolic blood pressure. Model 3: Model 2 + adjusted for triglyceride

*B* nonnormalized coefficient; *95% CI* 95% confidence interval

*P<0.05 **P<0.01 ***P<0.001

## Discussion

The present study showed that 82.2% CAPD patients developed gastrointestinal symptoms. The three most common gastrointestinal symptoms were dyspepsia, constipation and abdominal pain. Higher glycosylated hemoglobin, higher score of depression, lower urine output, diastolic blood pressure, score of IADLs and more amount of pills per day were independently associated with gastrointestinal symptoms of PD patients. Higher score of dyspepsia and eating dysfunction were independently associated with lower scores of QOL and physical component scale.

The prevalence of gastrointestinal symptoms in PD patients was high in this study. More than half of PD patients suffered from dyspepsia, and about a third of patients suffered from constipation and abdominal pain. These results were consistent with previous studies [2–5]. Salamon et al [2] found that 85% PD patients reported at least one gastrointestinal symptom which was defined as nausea, vomiting, bloating, early satiety, diarrhea, heartburn, fatigue, and weight changes. Dong et al [5] reported that 61.6% PD patients appeared at least one gastrointestinal symptom assessed by GSRS, and the prevalence of eating dysfunction, reflux and indigestion was 44.2%, 32.7% and 32.7%, respectively.

There might be multiple reasons for the high incidence of gastrointestinal symptoms in PD patients. To our knowledge, this study was the first one to show an association between glycosylated hemoglobin and gastrointestinal symptoms in PD patients. Schvarcz [23] and Kim [24] found that glycosylated hemoglobin level was associated with the gastrointestinal symptoms in diabetic patients. Based on large population studies, Bytzer et al [25, 26] raised the hypothesis that poor glycemic control by itself was a major cause of chronic gastrointestinal symptoms. Our results also found that depression was independently associated with gastrointestinal symptoms in PD patients. Chong et al [27] reported that psychosomatic symptoms such as anxiety, backache, depression, headache and insomnia were correlated significantly with gastrointestinal symptoms in hemodialysis patients. Kahvecioglu et al [28] found that depression was more frequent in PD patients with irritable bowel syndrome compared with those without. Lower urine output was independently associated with gastrointestinal symptoms of PD patients in this study. Dong et al [5] reported that residual renal Kt/V was negatively correlated to gastrointestinal symptoms of PD patients. It was suggested that residual renal function might play an important role on gastrointestinal symptoms in PD patients. They also [5] found that PD patients took an average of 15 pills a day, and the amount of pills daily intake was positively correlated with gastrointestinal symptoms in PD patients. Our results were similar to this report. Although we found that lower diastolic pressure and IADLs were independently associated with gastrointestinal symptoms in PD patients, the related mechanism was not yet clear. In addition, this study didn’t confirm the relationship between the dwell volume of dialysate and gastrointestinal symptoms. The main reason might be that 98.3% CAPD patients in this study were indwelled in the abdominal cavity with 2 liters of dialysate.

Only a few studies investigated the association of gastrointestinal symptoms with QOL in dialysis patients. Strid et al [4] found that gastrointestinal symptoms were negatively correlated with psychological general well-being in chronic renal failure patients. Zhang et al [14] reported that dialysis patients with constipation had significant lower mean physical component scale and mental component scale score than the non-constipation group. Although these studies found the correlation between gastrointestinal symptoms and QOL in dialysis patients, they did not perform multivariate analysis to adjust for other confounding factors. Using multiple linear regression analysis, our results confirmed that dyspepsia and eating dysfunction were independently associated with worse QOL and physical health in PD patients. It indicated that we should pay more attention to gastrointestinal symptoms to improve the QOL in PD patients.

The strength of this study was the relatively big number of samples, which may influence the power of statistical tests. But there were some limitations in this study. Firstly, the patients were enrolled in a single PD center, it was impossible to eliminate patient selection bias completely. Secondly, due to the cross-sectional nature of the study, it could not infer a causal relationship between gastrointestinal symptoms and other variables.

## Conclusions

This cross-sectional study demonstrated that 82.2% CAPD patients developed at least one gastrointestinal symptom. Glycemic control, depression, blood pressure, urine output, activity of daily life and total amount of pills were associated with gastrointestinal symptoms of PD patients. Moreover, gastrointestinal symptoms were significantly associated with QOL of PD patients.

## Data Availability

Not applicable.
